# Prevalence and clinical characteristics of Norwegians who report persistent health complaints attributed to tick bites or tick-borne diseases

**DOI:** 10.1186/s12879-025-12182-w

**Published:** 2025-11-27

**Authors:** Audun Olav Dahlberg, Audun Aase, Harald Reiso, Hanne Quarsten, Øivind Øines, Rune Midgard

**Affiliations:** 1 Norwegian University of Science and Technology, Trondheim, NO-7491 Norway; 2 Department of Neurology, Møre and Romsdal Hospital Trust, Molde, NO-6412 Norway; 3 Department of Method Development and Analytics, Norwegian Institute of Public Health, Oslo, NO-0213 Norway; 4 Norwegian National Advisory Unit on Tick-borne Diseases, Sørlandet Hospital Trust, Post-box 783, Arendal, NO-4809 Norway; 5 Department of Medical Microbiology, Sørlandet Hospital Trust, Kristiansand, NO-4615 Norway; 6 Norwegian Veterinary Institute, Ås, NO-1433 Norway; 7 Molde Hospital, Department of Neurology, Møre and Romsdal Hospital Trust, Parkvegen 84, Molde, 6412 Norway

**Keywords:** Persistent health complaints, Population prevalence, Tick bites, Tick-borne diseases, Seroprevalence, PCR

## Abstract

**Background:**

Persistent symptoms attributed to tick bites or tick-borne diseases are poorly understood. We estimate regionally adjusted prevalence of persistent symptoms, investigate seroprevalence (IgG) and ongoing infections, and examine associated demographic and clinical factors.

**Methods:**

Persons aged 18 years or older with persistent symptoms lasting six months or more attributed to tick bites or tick-borne diseases, were recruited into a nationwide cross-sectional study. Demographic data were recorded. Medical records were collected (February 2020 - April 2022) and reviewed for tick bites, tick-borne infections, antibiotic treatment, and clinical findings. Outcome measures included somatic symptoms (PHQ-15), fatigue (Fatigue Severity Scale), physical health (RAND-36), and affective symptoms (HAD Scale). Laboratory assessments included polymerase chain reaction (PCR) analysis of blood samples for *Borrelia burgdorferi* (Bb) and other known tick-borne pathogens, along with IgG antibody detection.

**Results:**

The highest prevalence of persistent symptoms attributed to tick bites or tick-borne diseases was found in southwestern Norway (0.152–0.155%); the lowest was in the north (0.033%), which also had significantly lower *Bb*-IgG seroprevalence (15.4% compared to the national average 37.5%). Symptom persistence was not associated with confirmed tick exposure or tick-borne infection. Somatic symptoms were associated with low physical activity and comorbidity. Fatigue and poor physical health were strongly associated with underemployment. Fatigue was also associated with depressive symptoms, low activity, sick leave, and comorbidities.

**Conclusions:**

Persistent symptoms were most prevalent in tick-endemic regions but were not associated with prior tick exposure or tick-borne infections. Symptom burden was primarily associated with comorbidities, especially physical inactivity and underemployment.

**Clinical trial number:**

Not applicable.

**Supplementary Information:**

The online version contains supplementary material available at 10.1186/s12879-025-12182-w.

## Background

Persistent health problems attributed to Lyme borreliosis (LB) remain controversial [1]. A recent study estimated the overall prevalence of such complaints in the Norwegian general population to be 0.15% [2]. Persistent symptoms after standard antibiotic treatment have a substantial impact on disability-adjusted life years (DALY) [3]. Long-lasting unexplained symptoms attributed to tick bites are hypothesized to be due to treatment failures or persistent infections caused by *Borrelia burgdorferi* sensu lato (*Bb*) or other tick-borne pathogens. These uncertainties, both among patients and doctors, have led to the idea that patients with medically unexplained symptoms (MUPS) [4] may suffer from a condition known as Chronic Lyme disease (CLD).

The term CLD is confusing, as some argue it reflects ongoing long-lasting tick-borne infection despite the lack of objective clinical or laboratory findings [5]. The International Lyme and Associated Diseases Society (ILADS) has attempted to define CLD, but it encompasses a broad spectrum of symptoms and lacks precise evidence of tick-borne infections [5, 6]. The term CLD was previously used to describe objectively verified persistent infections, but is now more appropriately referred to as late manifestations, according to the Infectious Diseases Society of America (IDSA) [7]. Case reports have shown that most patients diagnosed with CLD do not meet evidence-based diagnostic criteria for LB [8, 9]. Consequently, some advocate antibiotic treatment for MUPS often for several months, with substances that have not been adequately tested for efficacy [10–12].

A more comprehensive understanding of the clinical conditions manifesting as persistent symptoms attributed to tick bites or tick-borne diseases is needed. While the prevalence of persistent symptoms and their determinants have recently been evaluated in the Netherlands [13–15], adjusted national and regional prevalence estimates of persistent symptoms attributed to tick bites or tick-borne diseases, their association with demographic and clinical factors, and corresponding patient reported outcome measures (PROMs) have not been systematically studied.

In this study, we aim to estimate the national and regional prevalences of persistent health complaints attributed to tick bites or tick-borne diseases, the seroprevalence of antibodies against tick-borne pathogens, and the detection of possible on-going tick-borne infections by polymerase chain reaction (PCR). While our previous study [2] reported national prevalence, the current study provides regional population prevalence and seroprevalence estimates, compares high- versus low-endemic regions, validates self-reported symptoms, diagnoses, and treatments via medical records, and incorporates PCR data.

## Materials and methods

### Recruitment strategies

The study includes three cohorts recruited via Short Message Service (SMS), a public invitation, or through general practitioners (GPs). The inclusion and recruitment procedures, as well as general characteristics of these cohorts, have been described previously [2]. In that study, all three cohorts were analysed together; here, we focus specifically on differences between high- and low-endemic regions, defined by incident LB data from the Norwegian Surveillance System for Communicable Diseases (MSIS) and county population numbers from the Norwegian National Population Registry (NPR). Lyme borreliosis occurs most frequently along the Norwegian littoral, extending north to southern Nordland.

Between 5,000 and 10,000 individuals were invited from each of the eleven low-endemic counties, while 20,000–25,000 were invited from each of the eight high-endemic coastal counties (Vestfold–Møre og Romsdal), which consistently show the highest incidence. This selection reflects both incidence patterns and demographic variation. A detailed county-level overview for 2017, when the sample was planned, is provided in the supplementary material (Table S1). Region classification was available only for the SMS cohort. Participants had blood drawn by their GP.

Based on NPR and MSIS data, we defined the 11 low-endemic counties as Østfold, Oslo, Hedmark, Akershus, Oppland, Buskerud, Nord-Trøndelag, Sør-Trøndelag, Nordland, Troms, and Finnmark. The high-endemic regions included Møre og Romsdal, Rogaland, Vest-Agder, Aust-Agder, Telemark, Vestfold, Hordaland, and Sogn og Fjordane.

For geographical analyses, we also grouped the 19 counties into five natural regions:

- Northern Norway: Nordland, Troms, and Finnmark.
- Central Norway: Nord-Trøndelag, Sør-Trøndelag and Møre og Romsdal.
- Western Norway: Rogaland, Hordaland and Sogn og Fjordane.
- Southern Norway: Vest-Agder, Aust-Agder, Telemark, Vestfold, and Buskerud.
- Eastern Norway: Østfold, Oslo, Hedmark, Akershus, and Oppland.

Buskerud was classified as part of Southern Norway and Møre og Romsdal as part of Central Norway. Buskerud includes both inland areas with lower tick exposure and a coastal zone, whereas Møre og Romsdal is considered highly endemic. For analyses, counties were dichotomized into high- and low-endemic groups (Table S1). Comparisons between high- and low-endemic areas were therefore based on county-level classification, independent of the five-region grouping.

### Clinical variable assessment

We recorded demographic and clinical variables including Body Mass Index (BMI), comorbidities, concomitant medications, experiences with public and alternative health care, employment and sick leave status, and long-term treatment in public and alternative healthcare systems. Underemployment refers to people not in full employment. Patient reported outcome measures (PROMs) included the Patient Health Questionnaire-15 (PHQ-15) [16], the Fatigue Severity Scale (FSS) [17] and the RAND-36 health-related quality of life (HRQoL) survey. The RAND-36 provided an aggregated physical component summary (PCS) score. We also recorded the Hospital Anxiety and Depression Scale (HADS) [18]. Further details on the questionnaires are available in a previous publication [2]. Participants were offered the option to submit their medical records through a secure online solution or by regular mail for further review by three experienced physicians. Details on antibiotic treatment were provided, including whether antibiotic treatment was prescribed or not, the time since the last treatment, the latency of treatment in weeks, the number of treatments against LB, the duration of treatment, the effect of treatment and the type of specific antibiotic treatment. Information on verified tick bites, tick bite count, confirmed tick-borne disease before persistent symptoms, erythema migrans (EM), lymphocytoma, disseminated borreliosis (neuroborreliosis, Lyme arthritis and carditis, multiple EM, acrodermatitis chronica atrophicans), tick-borne encephalitis (TBE), symptoms attributed to a possible tick-borne disease and findings from cerebrospinal fluid was collected.

The medical records were collected between February 2020 and April 2022.

### Serological outcomes

IgG antibodies detected in this study included those against *Borrelia burgdorferi* sensu lato, Tick-borne encephalitis virus (TBEV), *Francisella tularensis*, *Coxiella burnetii*, *Anaplasma phagocytophilum*, *Babesia* spp., *Bartonella* spp., and *Rickettsia* spp. (Supplementary material, serology).

### DNA sampling and Preparation

DNA was extracted from EDTA-anticoagulated blood samples collected from study participants. Specifically, samples were collected from 101 persons recruited through public invitation and GPs, and from 285 persons recruited via SMS (Supplementary material).

### Detection of active infections

Real-time PCR for the detection of *B. burgdorferi* (2 assays), *B. miyamotoi*, *(A) phagocytophilum*, *Rickettsia* spp., *Neoehrlichia mikurensis* (2 assays), *F. tularensis*, *Bartonella* spp., and *C. burnetii* were applied. Multiple real-time PCR methods for detection of *Bab. microti*, *Bab. divergens*, and *Bab. venatorum* and other *Babesia* spp. as well as one traditional PCR used in combination with Sanger sequencing that would pick up multiple *Babesia* spp. if present, were applied to samples as specified (supplementary material, Table S12). PCR assays for *B. burgdorferi* and *N. mikurensis* were performed at accredited national reference laboratories in Norway and Sweden. Both assays are validated for human diagnostics and have demonstrated concordant results. All other PCR assays were performed at accredited laboratories using established, validated protocols for human diagnostics. Babesia PCR assays have not been systematically compared. Further technical details are provided in Supplementary material. No new gene sequences were generated in this study.

### Statistical analyses

#### Descriptive and univariate analyses

Standard descriptive statistics were presented to summarize demographic, clinical and psychosocial characteristics of the study population. Univariate analyses were performed to assess differences between groups, defined by endemic status (high vs. low), geographic region, and participants who delivered medical records or not. Outcomes included *Bb*-IgG and other tick-borne seroprevalence, as well as symptom and health measures (PHQ-15, FSS, RAND-36 (PCS), and HAD scores). Categorical variables were compared using chi-square tests or Fisher’s exact tests, and continuous variables using Student’s t-tests, Mann-Whitney U tests, as appropriate.

#### Population prevalence and seroprevalence

Population-level prevalence and seroprevalence of *Bb*-IgG were estimated using weighted logistic regression, adjusted for age (continuous) and sex. Information on nonparticipants (*n* = 269,608; invited but did not respond) was not analysed directly, but used to construct survey weights to adjust for response bias. For seroprevalence analyses, weights were based on incident LB cases reported to MSIS in 2017; for prevalence analyses, weights were based on population proportions. Weights were calculated by dividing the proportion of participants in each county by the proportion of the underlying population in the same strata. Weighted logistic regression models were fitted with group membership or *Bb*-IgG serology as the dependent variable, and region and endemic status of counties as covariates. Predicted probabilities were calculated for each individual and averaged across the sample to obtain adjusted national and regional prevalence estimates, which were visualized in tables and figures. Crude prevalences of other tick-borne microbes were compared between groups without adjustment.

#### Multivariable regression analyses of symptom, health, and work outcomes

Linear regression models were constructed for continuous outcomes (PHQ-15, FSS, RAND-36 (PCS)). Candidate predictors were selected using a two-step process (univariate analysis followed by age- and sex-adjusted linear regression), and variables meeting the selection criteria were included in stepwise/backward multiple regression models (entry α = 0.10, removal α = 0.15). Final models were adjusted for age and sex and calculated in a single block. Standardised coefficients (β) were used to assess predictor influence. Variables assessed are listed in the footnotes of Table 4. Model fit was evaluated with R^2^ and adjusted R^2^. Dichotomous work outcomes were analysed using stepwise/backward logistic regression on clinical variables selected from univariate and linear regression; significant predictors are reported as odds ratios (95% CI), with model fit evaluated using Cox & Snell and Nagelkerke R². Variables assessed are listed in the footnotes of Table 5.

#### Normative data

Questionnaire outcomes were compared with age- and sex-adjusted normative data: PHQ-15 (Swedish population) [19], RAND-36 and FSS (validated in Norway) [20, 21], and HADS (HUNT-3 cohort) [22].

#### Handling of missing data

Missing values were assumed missing completely at random (MCAR) and considered to have negligible impact on analyses, consistent with our previous study [2]. No systematic patterns of missing values were observed in the medical records.

#### Software and significance

All statistical analyses were performed using IBM SPSS Statistics version 29.0 and Stata version 18.0. A web-based calculator [23] was applied for comparison with normative data. Significance was set at α < 0.05.

## Results

### Study participants and demographics

A total of 470 participants were recruited: 14 via GPs, 93 by invitation, and 363 through SMS, as previously described [2]. The mean age was 54.4 years [53.0–55.8]. We received 169 of 470 (36.0%) medical records. Participants who provided medical records were older (58.6 years [56.4–60.7]) than those who did not (51.9 years [50.1–53.7]) with *p* < 0.001. Furthermore, a higher proportion of those with monthly net income > 20,000 NOK provided medical records (*p* = 0.016). A higher proportion of those who provided medical records were from high-endemic areas of tick-borne diseases (88.2%) compared to low-endemic areas (11.8%) (*p* = 0.033). A higher proportion of participants from high-endemic areas had lower educational levels (< 3 years after primary school) (29.9%) compared to low-endemic areas (12.2%) (*p* = 0.011). There were no significant differences in age, sex, BMI, physical activity, income, and employment status between those recruited from high-endemic areas and those from low-endemic areas. See Table 1 for details.

**Table 1:** Demographic data for Norwegians reporting persistent health complaints attributed to tick bites or tick-borne diseases, by high and low-endemic regions

	The total study population	The SMS-cohort (*N*)	High endemic (*N*)	Low endemic (*N*)	*p*-value
Participants	470	363	304 (83.7)	59 (16.3)	**< 0.001**
Age (mean, 95%CI)	54.4 (53–55.8) 392 (83.4%)	55.1 (53.5–56.7) 296 (81.5%)	55.7 (54.0–57.4) 250 (82.2%)	52.1 (47.6–56.6) 46 (83.1%)	0.113
Male	190 (45.2)	148 (45.7)	126 (46.2)	22 (43.1)	0.691
Norwegian	405 (96.4)	312 (86.0)	265 (97.1)	47 (92.2)	0.102
Education after primary school					
*< 3 years*	107 (25.7)	87 (27.2)	81 (29.9)	6 (12.2)	**0.011**
*3–6 years*	174 (41.8)	129 (40.3)	104 (38.4)	25 (51.0)	0.097
*> 6 years*	122 (29.3)	93 (29.1)	16 (32.7)	77 (28.4)	0.547
*Student*	13 (3.0)	11 (3.4)	9 (3.3)	2 (4.1)	0.679
Fully employed	123 (29.6)	97 (40.2)	81 (40.1)	16 (41.0)	0.914
Sick-listed	34 (8.2)	33 (10.3)	26 (9.6)	7 (14.3)	0.325
Fully disabled	73 (17.6)	56 (15.4)	48 (15.8)	8 (13.6)	0.664
Sick leave over a month last two years	187 (45.3)	135 (42.6)	108 (40.3)	27 (55.1)	0.054
Lives alone	83 (19.8)	66 (20.4)	53 (19.4)	13 (25.5)	0.323
Income (> 20.000 NOK)	381 (91.4)	293 (91.3)	248 (91.5)	45 (90.0)	0.784
BMI (mean, 95%CI)	26.7 (25.9–27.9)	26.9 (25.9–27.9)	27.2 (26.0–28.3)	25.2 (24.1–26.3)	0.143
Physical activity > 3 h per week	243 (57.9)	196 (60.5)	167 (61.2)	29 (56.9)	0.563

Continuous variables are presented as means with 95% confidence intervals, and categorical variables as counts and percentages. Statistical analyses were conducted using chi-square tests, Student’s t-tests, and Fisher’s exact tests, with *p*-values reported for differences between high- and low endemic regions

### Estimation of prevalence

#### Weighted national and regional prevalences (per 100000) of persistent symptoms attributed to tick bites or tick-borne disease in Norway

**Fig. 1:**
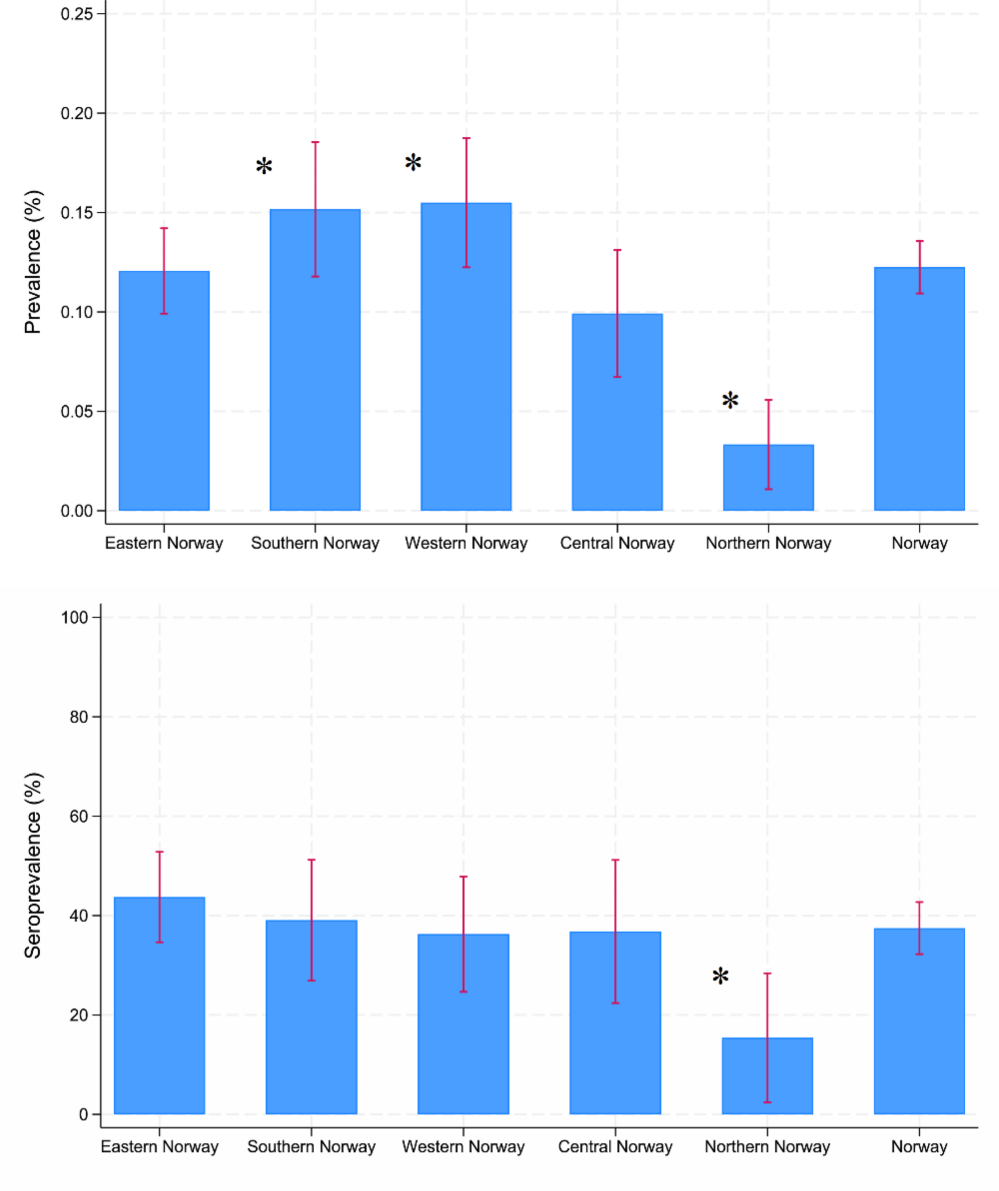
Population prevalences weighted by population proportions, and seroprevalences of *Bb*-IgG (%) weighted by incident Lyme borreliosis cases (2018) with 95% confidence intervals. Both population prevalences and seroprevalences were age- and sex-adjusted in this figure. (*) indicates a significant difference from Norway. Number of participants per region (percentage in parentheses): Eastern Norway: 41 (11.3%), Southern Norway: 172 (47.4%), Western Norway: 99 (27.3%), Central Norway: 46 (12.7%), Northern Norway: 5 (1.4%) and Norway: 363

The weighted and adjusted (age and sex) national prevalence of persistent symptoms attributed to tick bites or tick-borne disease was 122 (109–136). The regional adjusted and weighted prevalences compared to the national prevalence were as follows: Eastern Norway 121 (99–142, *p* = 0.501), Southern Norway 152 (118–186, *p* = 0.01), Western Norway 155 (122–187, *p* < 0.001), Central Norway 99 (67–131, *p* = 0.341), and Northern Norway 33 (11–56, *p* < 0.001). Adjusted prevalences were 94 (79–109) in low-endemic regions and 166 (142–190) in high-endemic regions, with the difference being significant (*p* < 0.001). The unadjusted weighted analyses were similar. See the histograms in Fig. 1. More detailed histograms are provided in Figure S1 and with details between regions in Table S3.

#### The crude, unadjusted, and weighted adjusted seroprevalence (%) of B. burgdorferi IgG and other tick-borne pathogens

We received blood samples from 385 of 470 (81.9%) of the included participants. There were no differences in serological results between those who provided their medical records and those who did not. The unadjusted crude seroprevalences by region for all tick-borne pathogens examined in this study were compared with national seroprevalences.

The crude analyses of all tick-borne pathogens examined are shown in Table 2.

**Table 2:** Crude Seroprevalence of tick-borne infectious agents in Norway by health region, and by high- and low-endemic areas (SMS-cohort)

Regions	Norway (*N* = 285)	Eastern (*N* = 30) *p*-value*	Southern (*N* = 135) *p*-value*	Western (*N* = 78) *p*-value*	Central (*N* = 38) *p*-value*	Northern (*N* = 4) *p*-value*	High endemic (*N* = 239)	Low endemic (*N* = 46)	*p*-value**
*Borrelia burgdorferi*	127 (44.6)	12 (40.0) 0.630	69 (51.1) 0.213	28 (35.9) 0.169	17 (44.7) 0.991	1 (25.0) 0.434	110 (46.0)	17 (37.0)	0.257
*Anaplasma phagocytophilum*	10 (3.5)	1 (3.3) 0.955	6 (4.4) 0.652	3 (3.8) 0.899	0 0.242	0 0.704	9 (3.8)	1 (2.2)	1
*Bartonella sp.*	3 (0.01)	0 0.956	1 (0.7) 0.170	1 (1.3) 0.06	1 (2.6) **0.008**	0 0.984	3 (1.3)	0	1
*Rickettsia sp.*	35 (12.3)	3 (10.0)	17 (12.6)	7 (9.0)	8 (21.1)	0	30 (12.6)	5 (10.9)	0.750
*Coxiella burnetii*	0	0	0	0	0	0	0	0	NA
Tick-borne encephalitis virus***	27 (9.5)	2 (6.7) 0.615	24 (17.8) **0.015**	1 (1.3) **0.017**	0 **0.048**	0 0.518	25 (10.5)	2 (4.3)	0.274
*Fransiscella tularensis*	17 (6.0)	0 0.168	8 (5.9) 0.968	6 (7.7) 0.586	3 (7.9) 0.586	0 0.864	16 (6.7)	1 (2.2)	0.325
*Babesia sp.*	19 (6.7)	2 (6.7) 1	9 (6.7) 1	6 (7.7) 0.758	2 (5.3) 0.743	0 0.593	16 (6.7)	3 (6.5)	1
PCR									
*N. mikurensis*	11 (3.9)	0 0.272	8 (5.9) 0.359	2 (2.6) 0.587	1 (2.6) 0.692	0 0.688	10 (4.2)	1 (2.2)	1

* *P*-values indicate statistically significant differences between Norway overall and the different regions

** *P*-values indicate statistically significant differences between high- and low endemic regions

***Among those unvaccinated against TBEV, all were seronegative for TBEV-IgG, except in the southern region where 5 out of 93 (5.4%) were seropositive

The adjusted and weighted national seroprevalence of *Bb*-IgG was 37.5% (32.2–42.7). The regional adjusted and weighted prevalences compared to the national prevalence were as follows: Eastern Norway 43.7% (34.6–52.8, *p* = 0.078), southern Norway 39.1% (26.9–51.2, *p* = 0.649), western Norway 36.3% (24.7–47.9, *p* = 0.957), central Norway 36.8% (22.4–51.2, *p* = 0.899), and northern Norway 15.4% (2.4–28.4, *p* = 0.012). The adjusted and weighted prevalences were 34.6% (27.8–41.4) in low-endemic regions and 41.8% (33.2–50.5) in high-endemic regions, but the difference was not significant (*p* = 0.130). The unadjusted and weighted national seroprevalences were largely similar.

See histograms in Fig. 1. More detailed histograms are provided in Figure S1 and with details between regions in Table S3.

### Detection of tick-borne disease pathogen DNA

Of the 386/470 tested blood samples, 12 had positive PCR for *N. mikurensis* and one of them was lost to follow-up. The remaining 11 persons with positive PCR of *N. mikurensis* have been described in a separate paper [24]. All other pathogens tested were negative (*B. burgdorferi*,* B. miyamotoi*,* A. phagocytophilum*,* Rickettsia* spp., *F. tularensis*,* Bartonella*,* C. burnetii*,* Bab. microti*, *Bab. divergens*, *Bab. venatorum* and other *Babesia* spp.) (supplementary material).

### Clinical data from medical records

#### Tick bites, symptoms attributed to lyme disease, diagnosis, and treatment

The most prominent symptoms were fatigue (*n* = 33, 19.5%), musculoskeletal problems (*n* = 26, 15.4%) and pain (*n* = 18, 10.7%). Of the 169 participants, fourteen (8.3%) had a confirmed tick-borne disease before persistent symptoms, while 102 (60.4%) had a verified and treated LB. A verified tick bite was found in 59 (34.9%), while 47 (27.8%) have been diagnosed with EM or lymphocytoma, and 38 (22.5%) had disseminated borreliosis. Neuroborreliosis was verified by lumbar puncture in 17 (10.1%) and tick-borne encephalitis in 3 (1.8%). Regarding antibiotic treatment, 12/53 (22.6%) had treatment latency for more than six weeks, 38/144 (26.4%) had two or more antibiotic treatments, and 28/169 (16.6%) had treatment of four weeks or more. See Table S8 for details. Symptoms and cognitive difficulties attributed to LB were collected as shown in Table S9.

#### Antibiotics

Regarding EM and lymphocytoma, doxycycline was prescribed for 17/47 (36.2%), oral penicillin for 12/47 (25.5%), intravenous penicillin for 2/47 (4.3%), and intravenous ceftriaxone for 1/47 (2.1%). Persons with disseminated borreliosis, doxycycline was prescribed in 24 of 38 cases (63.2%), oral penicillin in 8/38 (21.1%), intravenous penicillin in 5/38 (13.2%), and intravenous ceftriaxone in 3/38 (7.9%). For treated persons with noted improvement, no change, or worsening of symptoms, improvement was verified in 9/11 (81.8%) after oral doxycycline for EM or lymphocytoma, 10/13 (76.9%) for disseminated borreliosis, and 6/7 (85.7%) for verified neuroborreliosis. Symptomatic improvement was observed in 36/45 (80%) for oral doxycycline, 19/20 (95%) for oral penicillin, and 5/6 (83.3%) for intravenous ceftriaxone. With a treatment latency of more than six weeks, 7/9 (77.8%) showed an improvement in symptoms after treatment. Among those with confirmed tick-borne disease prior to persistent symptoms, all showed improvement after treatment; see Tables S10 and S11 for details.

### Treatment experiences

For the total study population, 285/411 people (69.3%) reported being satisfied with follow-up in public health institutions, while 87/140 people (62.1%) were satisfied with follow-up in alternative institutions. A proportion of 23.1% of the study population had been diagnosed with LD outside the Norwegian public healthcare system and 34.9% had received long-term antibiotic treatment for more than 4 weeks. There were no significant differences when comparing high and low-endemic areas. However, a higher proportion (80.5%) of those who submitted their medical records, compared to those who did not (62.6%), were satisfied with the treatment they received from the public healthcare system (*p* < 0.001). Furthermore, persons who submitted medical records were less frequently diagnosed by alternative laboratories in Norway or abroad. (15.2% vs. 28.0%, *p* = 0.003). See Table 3 for details.

**Table 3:** Experiences with diagnoses, and treatment in public and alternative healthcare systems in Norway and abroad. The number (N) and given per cent (%)

	High endemic (*N*)	Low endemic (*N*)	The SMS-cohort (*N* = 363)	The total study population** (*N* = 470)	*p*-value***
Satisfied with public health care	209 (78.6)	42 (85.7)	251 (79.7)	285 (69.3)	0.253
Satisfied with alternative health care	22 (57.9)	5 (83.3)	27 (61.4)	87 (62.1)	0.380
Diagnosis of LD outside the Norwegian public health care system; Norway or abroad	42 (15.6)	7 (14.3)	49 (15.4)	96 (23.1)	0.821
Detection of tick-borne pathogens alternative institution abroad					
*Germany*	1	0	1	15	
*Hungary*	0	0	0	4	
*Poland*	0	0	0	2	
Pathogen detected in alternative laboratory*					
*B. burgdorferi sp.*	16	2	18	30 (5/24)	
*Babesia sp.*	2	1	3	8 (1/2)	
*Bartonella sp.*	2	0	2	6 (0/5)	
*Anaplasma sp.*	0	0	0	1 (0/1)	
*Fransiscella sp.*	0	1	1	1 (0)	
Long term antibiotic treatments > 4 weeks in Norway or abroad	76 (28.3)	17 (34.7)	93 (29.3)	144 (34.9)	0.362

*The fraction (in parentheses) shows the number of seropositive cases detected by our laboratory among those with test results for the same agent from alternative laboratories. Data on the type of tests from the alternative laboratories are missing

**All recruitment methods: via GPs, invitation and through SMS

***High vs. low endemic region

### PROMs

There were no significant differences between participants from high- and low endemic areas in severe fatigue (FSS > 4), more than moderate symptom burden (PHQ-15 > 10), or reduced physical health (PCS < 50). In the total study population (*n* = 470), 79.8% had reduced physical health (PCS < 50), while the proportions with severe fatigue and more than moderate symptom burden have been reported elsewhere [2]. Most participants, 399/410 (97.3%), had either high symptom burden, reduced physical health, or severe fatigue. In adjusted analyses, there was a higher degree of anxiety (*p* = 0.027) and reduced physical health (*p* = 0.049) among those who did not submit medical records. In the SMS-cohort alone, there were more somatic symptoms (*p* = 0.042) and a higher degree of anxiety (*p* = 0.009) among those who did not present medical records. Furthermore, there was a higher degree of anxiety (*p* = 0.037) in low-endemic regions compared to high-endemic regions. In the further adjusted analyses, there were no significant differences between high and low endemic regions and between those who submitted and did not submit their medical records in the other PROMs analysed. The average scores for PHQ-15, PCS, FSS, as well as HADS depression and anxiety, were all significantly worse in cases compared to normative data (*p* < 0.001). Refer to Tables S4–S6 for detailed parameter estimates, and Table S7 for comparisons with normative data.

### Clinical and health-related outcomes

The variables included in the stepwise analysis adjusted for age and sex are described in the footnotes of Table 4. For somatic symptoms (PHQ-15), the most significant determinants were physical activity less than 3 hours per week, *Bb*-IgG seronegativity, and the presence of one or more comorbidities. Underemployment was most strongly associated with reduced physical health (PCS) and increased fatigue (FSS). Additional variables linked to increased fatigue included physical activity less than 3 hours per week, more than borderline depression, and antibiotic treatment for more than 4 weeks. Regarding sick leave and employment, the variables most strongly associated with underemployment were more than one comorbidity, more than borderline depression, a net income below 20,000 NOK per month, decreased PCS, and female sex. Long-term sick leave (> 1 month) within the past two years was associated with severe fatigue (FSS > 4), age 18–49 years, and lower PCS scores. The full results are shown in Table 5, with details of included variables provided in the footnotes.

**Table 4:** Risk factors associated with somatic symptoms (PHQ-15), physical health (PCS) and fatigue (FSS)

PROMs	PHQ-15*			PCS**			FSS***		
Parameters	B	Standardized beta (β)	*p*-value	B	Standardized beta (β)	*p*-value	B	Standardized beta (β)	*p*-value
Physical activity > 3 h per week	**-3.687**	**-0.302**	**0.005**	-	-	-	**-0.8545**	**-0.238**	**0.015**
Bb-IgG	**-3.448**	**-0.284**	**0.006**	-	-	-	-	-	-
One or more comorbidities	**3.582**	**0.269**	**0.014**	-	-	-	-	-	-
Male	-2.280	-0.190	0.067	0.678	0.045	0.643	-0.242	-0.068	0.471
HADS Depression > 8	2.052	0.152	0.128	-	-	-	**0.899**	**0.220**	**0.019**
Net income > 20.000 NOK	-3.998	-0.151	0.128	-	-	-	-	-	-
Underemployed	1.477	0.120	0.294	**-4.277**	**-0.281**	**0.006**	**1.070**	**0.294**	**0.003**
Age 18–49 years	-0.019	-0.001	0.988	-1.478	-0.090	0.343	0.069	0.018	0.851
Verified and treated LB	-	-	-	2.664	0.174	0.076	-	-	-
HADS Anxiety > 8	-	-	-	2.545	0.146	0.131			
Long term antibiotic treatment > 4 weeks	-	-	-	-	-	-	**0.744**	**0.204**	**0.031**

Demographics like age (categorized as 18–49 and 50 and older), sex, living status, net income over 20,000 NOK, employment status (excluding age pensioners and students), education more than six years from primary school, one or more comorbidities and physical activity were assessed. Variables related to history of LB were also considered: High- or low-endemic regions, verified tick bite or not, two or more tick bites, serology (IgG), long-term treatment more than 4 weeks or not, treatment latency over 6 weeks, the number of antibiotic treatments (two or more treatments or not), EM or lymphocytoma (stage I LB), disseminated borreliosis (stage II LB and TBE) and late borreliosis with ACA (stage III LB), verified and treated LB, verified neuroborreliosis and verified tick-borne disease prior to the onset of persistent symptoms. Other variables as borderline depression (HAD depression > 8), borderline anxiety (HAD anxiety > 8) and BMI were also assessed. Empty cells (-) indicates that the variables were excluded in the stepwise and backward regression analyses. R^2^ = 0.419*, 0.181**, 0.272***. Adjusted R^2^ = 0.348*, 0.137**, 0.224***. B denotes the unstandardized regression coefficient. A higher absolute standardized β indicates greater influence on the outcome variable (shown in bold)

**Table 5:** Risk factors for sick leave and underemployment

Parameters	The risk of underemployment*				The risk of long-term sick leave (>1 month) **			
	B	OR(eB)	*p*-value	95% CI	B	OR(eB)	*p*-value	95% CI
FSS > 4	-	-	-	-	**1.257**	**3.516**	**0.001**	**1.621–7.626**
More than one comorbidity	**1.432**	**4.2**	**< 0.001**	**2.1–8.3**	-	-	-	-
HADS depression > 8	**0.830**	**2.3**	**0.022**	**1.1–4.7**	-	-	-	-
Net income > 20.000 NOK per year	**-1.797**	**0.166**	**0.028**	**0.033–0.828**	-	-	-	-
PCS	**-0.072**	**0.931**	**< 0.001**	**0.897–0.966**	**-0.48**	**0.953**	**0.002**	**0.925–0.982**
Male	**-1.139**	**0.320**	**< 0.001**	**0.176–0.584**	-0.373	0.689	0.127	0.427–1.112
Age 18–49 years	-0.410	0.664	0.181	0.364–1.210	**0.947**	**2.578**	**< 0.001**	**1.577–4.214**

Univariate analyses assessed the impact of Lyme disease. Clinical variables on sick leave and employment, including EM and lymphocytoma, disseminated borreliosis and ACA, antibiotic treatment history (time since last treatment > 1 year, treatment delay > 6 weeks, ≥ 2 treatments), medical record availability, confirmed and treated LB, and high- or low endemic region. No significant associations with sick leave and underemployment were observed. Key predictors identified in stepwise/backward multivariable regression were evaluated via binary logistic regression with likelihood ratios to estimate risks of sick leave and underemployment. These included physical activity, borderline depression (HADS depression > 8), borderline anxiety (HADs anxiety > 8), long-term treatment (> 4 weeks), Bb-IgG serostatus, age (18–49, 50+), sex, comorbidities, severe fatigue (FSS > 4), moderate or greater somatic symptoms (PHQ-15 > 10), physical health (PCS), net income > 20,000 NOK, and education. *Model summary: -2LL = 269.187 R^2^ = 0.254 (Cox & Snell), 0.347 (Nagelkerke), ** Model summary: -2LL = 404.314 R^2^ = 0.168 (Cox & Snell), 0.225 (Nagelkerke). Long-term sick leave = absence > 1 month (assessed over the past two years). Empty cells (-) indicates that the variables were excluded in the stepwise and backward regression analyses. B is the change in log-odds of the outcome per one-unit increase in the predictor

For comorbid diseases and concomitant medications, there was a lower proportion of psychiatric disease among participants who provided their medical records (*n* = 12, 8.6%) compared with those who did not (*n* = 45, 16.1%, *p* = 0.034). No significant differences in comorbid diseases or concomitant medications were observed when comparing high- and low-endemic areas (Table S2).

## Discussion

We report that the southwestern regions of Norway showed the highest prevalence of persistent symptoms attributed to tick bites (152–155 per 100,000), while the northern areas had the lowest prevalence (33 per 100,000). The northern areas had a significantly lower *Bb*-IgG seroprevalence of 15.4% compared to 37.5% nationally, while the other regions exhibited similar rates. Based on medical records, exposure to verified tick bites and tick-borne diseases was not associated with decreased health outcomes. Furthermore, the somatic symptom burden was most strongly associated with physical inactivity, while for severe fatigue and reduced physical health, underemployment was the most significant factor.

By summarizing the number of reported cases of LB in 2018 (8/100,000) from MSIS (EM not reported) with an estimated annual incidence of EM of 0.15% in Norway [25], the total estimated annual incidence of LB becomes approximately 0.16%. Assuming that up to 20% of LB patients develop persistent symptoms [26], this corresponds to an estimated annual incidence of approximately 0.031% for post-treatment or persistent Lyme-related symptoms. Compared to the national point prevalence of 0.12% for persistent symptoms attributed to tick bites found in our study, this may suggest that symptoms persist over longer time in some persons. However, we cannot rule out the possibility that other contributing factors, such as comorbidities [2], play a role.

The higher prevalence of persistent symptoms in the southwestern and highly endemic areas cannot be explained solely by increased tick exposure, as we found no significant differences in confirmed tick bites, *Bb*-seroprevalence, or confirmed tick-borne diseases between high- and low-endemic areas. In our previous study [2], 35% self-reported a tick bite in the past year and 73% multiple lifetime bites, with no difference between symptomatic participants and controls. Since many bites go unnoticed, self-reported exposure cannot imply causality. While self-reported bites may not reliably indicate exposure, even confirmed bites and diagnoses did not explain regional differences. However, national migration may explain the similarities in seroprevalence of tick-borne pathogens between the regions. Additionally, the seroprevalence of *Bb* in Northern Norway remains uncertain due to a low number of blood samples. An exception is TBEV, with a higher seroprevalence in the southern region (17.4%, *p* < 0.015). Since only 5.4% were unvaccinated, natural TBE infection may explain only some long-term symptoms in this area. The incidence of LB and prevalence of *Ixodes ricinus* are highest in southern and western Norway [27]. More protective behaviour and perceived risk have been found among persons living in high-endemic areas [28, 29]. Increased awareness may have led persons to attribute persistent symptoms to tick bites, even when other underlying causes, such as comorbidities, may have been responsible for their symptoms [30]. However, *Bb*-IgG-seroprevalence in our total sample was clearly higher than both the nationally adjusted seroprevalence in persons over 20 years of age [31] and findings from a study conducted in a high-endemic area [32]. Among those diagnosed with LB by alternative laboratories, only 20.8% were confirmed by our serological tests (*Bb*-IgG), closely matching *Bb* seroprevalence observed in the high-endemic Søgne cohort [32]. The low confirmation rate may reflect misdiagnosing due to the use of inappropriate diagnostic tests or a decline in *Bb*-specific IgG antibody levels over time. Although IgG antibodies can persist for many years following treatment, they may eventually decline below detectable levels, especially when infections are treated early [33, 34]. However, it is likely that fewer persons than assumed have been infected with or exposed to *Bb*.

DNA from the tick-borne pathogen *N. mikurensis* was detected in low amounts by PCR in the blood of 12 persons; however, a follow-up study of 11 of these cases revealed no greater symptom burden compared to other participants with persistent symptoms [24]. None of the other eight tick-borne pathogens were detected by PCR in the blood samples. A positive PCR result generally indicates an active infection and is usually most sensitive for detecting tick-borne microbes during the early stages of infection [35–37]. *Borrelia burgdorferi* is rarely present in blood (except in early infection or immunosuppressed patients), so PCR is primarily used on synovial fluid or cerebrospinal fluid for diagnosing Lyme arthritis, neuroborreliosis, atypical EM, and acrodermatitis chronica atrophicans (ACA), whereas other tick-borne pathogens examined in our study typically circulate or multiply in blood cells and are more readily detected by PCR [33]. However, the results suggest that active infections from known tick-borne pathogens that were investigated are unlikely in these persons and raise the possibility of misdiagnosis, which may occur if clinical criteria are not rigorously applied or if unreliable diagnostic methods are used.

The most common clinical symptom found in medical records was fatigue (19.5%). The prevalence of severe fatigue in the Norwegian population is 2% [38], and the prevalence of chronic fatigue syndrome/myalgic encephalitis (CFS/ME) varies between 0.1 and 2.5% depending on the criteria used [39]. In a Scottish population study, the adjusted prevalence of long-COVID symptoms attributable to SARS-CoV-2 was estimated at 6.6% − 10.4%, while 40.2% of infected individuals reported tiredness six months after infection [40]. Although different methods underlie these figures, they show that fatigue is a common feature of these conditions.

Most participants in our study were satisfied with the treatment received from both public and alternative healthcare services. Those who submitted medical records and were satisfied with public healthcare used alternative laboratories less frequently compared to those who did not provide medical records. Some study participants may have hidden tests or lack documentation because they only used alternative care. Their symptoms may be different or vaguer, leading to further evaluation outside the public system. Although we do not know for sure about those who did not submit medical records, the majority of those who did submit records experienced symptom improvement after treatment. Tick exposure is high in our study population, but very few (14/169) had persistent symptoms potentially associated with tick bites or tick-borne infections.

Persistent symptoms attributed to tick bites have long been a controversial topic, eliciting strong opinions from both medical professionals and the public [10]. Hypotheses to explain this include tissue persistence of *Bb* (e.g. via biofilm [41]) and post-infectious or host-mediated sequelae [42]. A recent study indicate that *Bb* peptidoglycan can persist in discrete tissues and elicit systemic immune responses [43], whereas Dutch and American randomized controlled trials (RCT) have consistently failed to demonstrate benefit from prolonged antibiotic treatment [12, 44]. A Norwegian RCT further showed that standard antibiotic therapy for Lyme neuroborreliosis (LNB), consisting of two weeks of doxycycline, is sufficient [45]. These contrasting lines of evidence underpin the divergent recommendations of ILADS and IDSA guidelines for managing persistent symptoms following tick-bites and Lyme disease [6, 7]. A notable 34.9% of our study participants had undergone long-term antibiotic treatment abroad, but relatively few of them had received a Lyme diagnosis from alternative laboratories. Our findings indicate that long-term antibiotic treatment (> 4 weeks) is not associated with reduced fatigue in persons attributing persistent symptoms to ticks. On the contrary, increased fatigue was associated with such treatment, possibly reflecting a lack of therapeutic effect or that persons with more severe symptoms are more likely to receive prolonged treatment.

Misinformation from media and the internet [46] can lead to cognitive bias [47]. Cognitive mechanisms for functional disorders may involve the nocebo effect, body awareness, and negative expectations increasing anxiety and attention to one’s own symptoms [48]. An ‘infectious’ disease, like CLD, can also more readily reduce stigma [49, 50], thereby reinforcing the desire to obtain a treatable diagnosis. Comparatively, another study during the COVID-19 pandemic, found that persistent physical symptoms were more associated with the belief of being infected than with confirmed infection [51]. Furthermore, persistent physical symptoms are very common in the general population, with both symptom expression and underlying explanations being heterogeneous [52, 53].

A study found no differences in symptoms, laboratory results, or disease progression between a group with presumed post Lyme disease syndrome (PTLDS) and various groups without evidence of *Bb* exposure [54]. A previous Dutch study found an increased prevalence of persistent symptoms after treatment for LB, with fatigue being the most significant outcome [15]. Another Dutch study [13] identified several predictors for persistent symptoms following confirmed LB, including low baseline social and physical functioning, affective symptoms, negative illness perceptions, and fatigue. In a separate subgroup from the same study, comprising individuals with self-attributed persistent symptoms without clinical or laboratory confirmation, unemployment at baseline was the only significant factor. At the one-year follow-up, 80.7% met criteria for fatigue, cognitive impairment, or pain, consistent with our findings where 97.3% had severe fatigue, high symptom burden, or reduced physical health (overlapping but not identical symptom profiles). Another study found no signs of *Bb* infection after treatment but suggested that immune factors, such as elevated IL-23 and autoantibodies, may be relevant in some patients with post-treatment symptoms [55].

In our study population, fatigue was associated with low physical activity, depression, and reduced work participation. Increased somatic symptom burden correlated with decreased physical activity, *Bb* seronegativity, and comorbidities. Reduced physical health was associated with low work participation, which was further associated with comorbidities, depression, low income, and female sex. Notably, long-term sick leave showed the strongest associations with both fatigue and reduced physical health. Prior documented tick bites or tick-borne infections, based on medical records, were not associated with the health outcomes in adjusted analyses. Stepwise regression with PHQ-15, FSS and PCS as dependent variables, adjusted for age and sex, showed that psychosocial and functional factors had the strongest associations with symptom severity. While these factors cannot be used to directly confirm or refute the persistent infection hypothesis due to limitations in tick bite documentation, they underscore the importance of a multidisciplinary approach focusing on physical activity, mental health, work, and coping. Building patient trust through clear explanation of disease mechanisms remains crucial for effective long-term treatment alliances.

Our study population may share many phenotypic similarities with other related conditions, such as CFS/ME [56, 57]. One of the cardinal symptoms of CFS/ME is ‘post-exertional exhaustion,’ and in our study population, low physical activity was the variable most strongly associated with fatigue. The direction of this association remains unclear. Nevertheless, physical activity and cognitive behavioural therapy has been found to have an effect in CFS/ME [58] and long-COVID-19 [59], and it may be worthwhile to further investigate this in a population with persistent physical symptoms attributed to ticks. Furthermore, persons with unexplained physical symptoms often seek meaningful explanations, which may explain why many focus on ticks as a possible cause despite lacking clinical evidence. This underscores the need for better education and interdisciplinary dialogue about both tick-borne diseases and how patient perceptions shape their care.

This study has several limitations. The study is retrospective, and there are possibilities of misclassification and recall bias in the questionnaire responses, and possible exclusion of data from specialist healthcare services. Additionally, the records might be incomplete, with some information potentially lost due to changes of GPs. However, the records were directly provided by participants who requested copies from their GPs, suggesting they intended to submit the necessary information regarding various tick-borne infections. It is possible that some information was deliberately omitted, as only 36% of participants submitted their medical records. There may be selection bias in submitted medical records, with a slight overrepresentation of older, high-income individuals from highly endemic areas. Conversely, recruitment via SMS may be biased toward younger, healthier participants. PCR results depend on the availability of DNA/RNA in the sample, which varies with disease progression and tissue type, and can be affected by inhibitors that impede amplification. The *Babesia* PCR assays used have not been systematically validated on clinical human samples, so data on detection sensitivity and specificity are not available. The selection of DNA isolation kit and protocols is critical, as some pathogens may be difficult to access or may reside in different fractions of the sample than those typically processed. In some cases, pathogens may be present in such low numbers that host DNA dominates the extraction, potentially overwhelming the system and reducing sensitivity, unless enrichment procedures are implemented. Additionally, sample quality can be compromised by inadequate storage conditions or repeated freeze-thaw cycles, which may degrade nucleic acids and negatively influence PCR results. Therefore, negative PCR results should be interpreted with caution and considered alongside clinical findings and serology, as outlined above. However, the sample material in this study was processed and analysed with expertise, which strengthens the reliability of the findings. Review of medical records identified only 14/169 participants in whom other causes did not fully explain their symptoms, suggesting that persistent infection cannot be completely excluded in these few cases; however, immune status was unknown.

## Conclusions

We found the highest prevalence of persistent symptoms attributed to tick bites or tick-borne diseases in highly tick-endemic areas, mainly southwestern Norway. However, symptom persistence was not associated with a medical history of tick bites or evidence of tick-borne infections. Somatic symptoms correlated with low physical activity and the presence of comorbidities. Underemployment was most strongly associated with reduced physical health and increased fatigue. Other key factors associated with fatigue included low physical activity and depressive symptoms. Fatigue was also associated with an increased risk of long-term sick leave, whereas comorbidity was associated with underemployment. Future research should clarify mechanisms of symptom persistence, while clinical practice must focus on patient education, interdisciplinary dialogue, and tailored management strategies.

## Supplementary Information

Below is the link to the electronic supplementary material.

Supplementary Material 1

## Data Availability

The datasets generated and/or analysed during the current study are not publicly available due to privacy concerns and the potential for re-identification. However, they can be obtained from the corresponding author upon reasonable request.

## Abbreviations

ACA: Acrodermatitis chronica atrophicans
Bb: Borrelia burgdorferi sensu lato
CFS/ME: Chronic fatigue syndrome/myalgic encephalitis
CLD: Chronic Lyme disease
EM: Erythema migrans
FSS: the Fatigue Severity Scale
GDPR: General Data Protection Regulation
GP: General practitioner
HADS: The Hospital Anxiety and Depression Scale
HRQoL: Health-related quality of life
ILADS: The International Lyme and Associated Diseases Society
LB: Lyme borreliosis
LNB: Lyme neuroborreliosis
MCAR: Missing completely at random
MSIS: The Norwegian Surveillance System for Communicable Diseases
MUPS: Medically unexplained symptoms
NPR: The Norwegian National Population Registry
OR: Odds ratio
PCR: Polymerase chain reaction
PCS: Physical component summary
PHQ-15: The Patient Health Questionnaire-15
PROMs: Patient-reported outcome measures
RCT: Randomized controlled trial
PTLDS: Post Lyme disease syndrome
SMS: Short Message Service
TBEV: Tick-borne encephalitis virus
