# Supplementary material for: Prevalence and clinical characteristics of Norwegians who report persistent health complaints attributed to tick bites or tick-borne diseases
Source: BMC Infect Dis. 2025 Nov 27;25:1663. doi: 10.1186/s12879-025-12182-w (PMC12659467; doi:10.1186/s12879-025-12182-w)
Supplement: Supplementary file 1 — Supplementary Material 1 [file 12879_2025_12182_MOESM1_ESM.docx]

***Supplementary material:***

Manuscript Title: *Prevalence and clinical characteristics of Norwegians who report persistent health complaints attributed to tick bites or tick-borne diseases,* by Dahlberg et al.


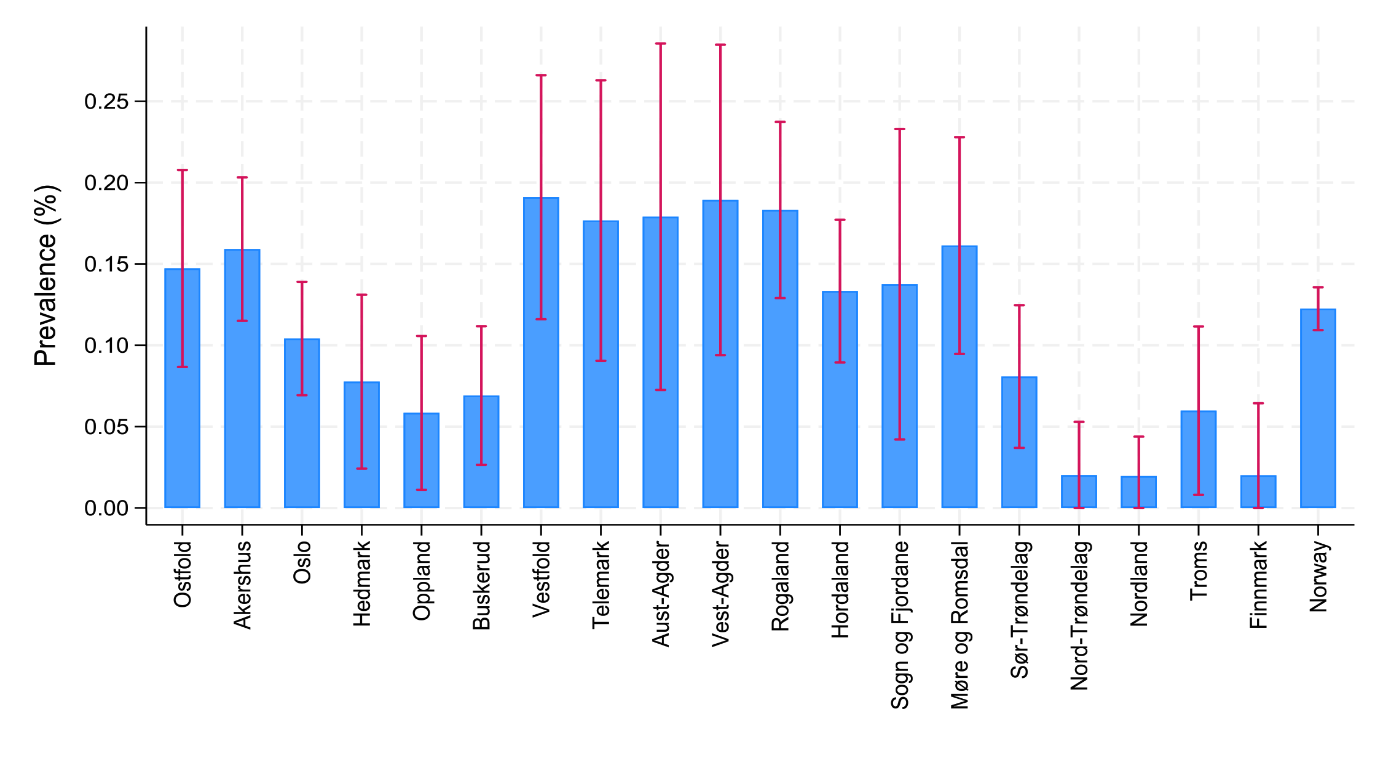

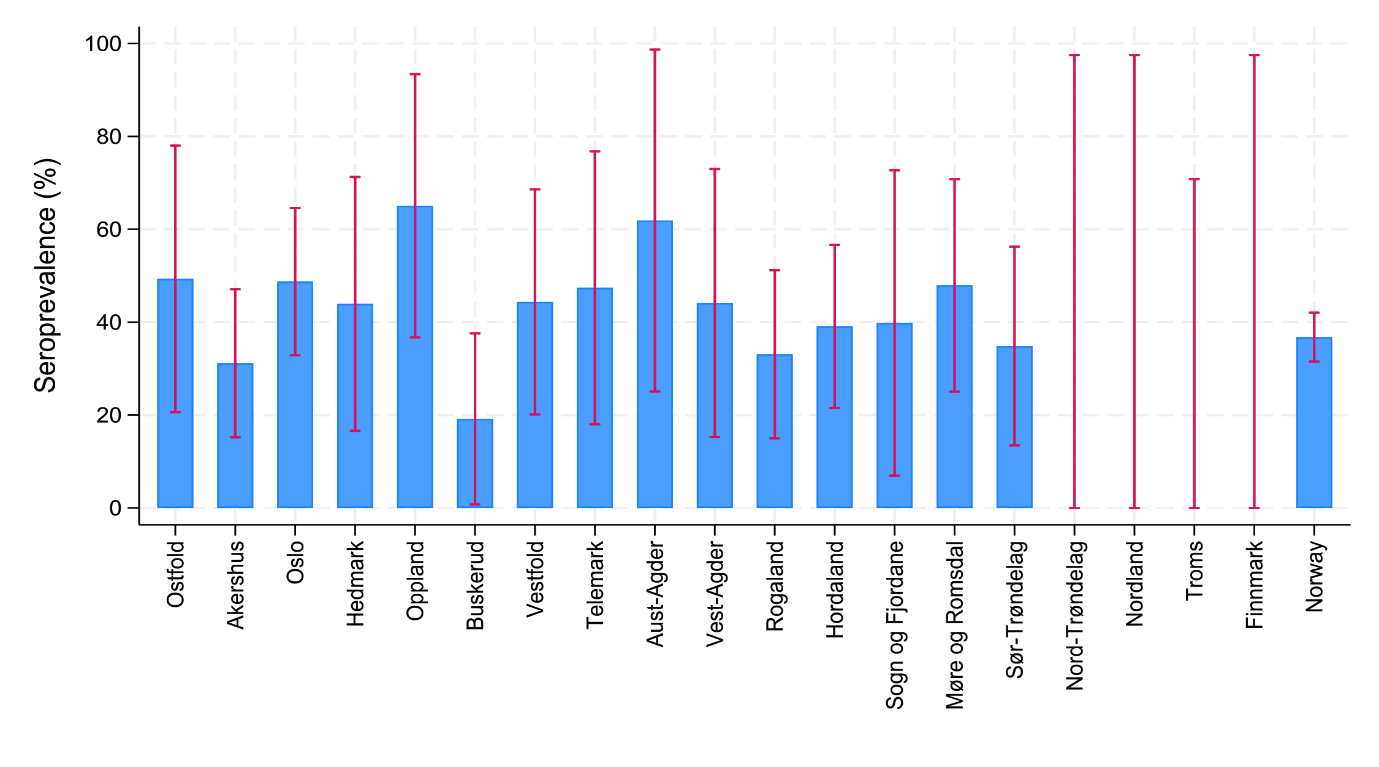


Figure S1 –Population prevalences weigthed by population proportions and seroprevalences of Bb-IgG (%)

weighted by incident Lyme borreliosis (2018) with 95% confidence intervals. Both population prevalences and

seroprevalences were age and sex adjusted.

| **Table S1.**  Sampled counties, 2017 Lyme borreliosis (LB) incidence, county population, and incidence per population including sampled participants. | | | | |
| --- | --- | --- | --- | --- |
| County * | Total LB 2017 | Population | Incident LB/100,000 people | Sample >= 18 years |
| Østfold | 26 | 292893 | 9 | 10000 |
| Akershus | 43 | 604368 | 7 | 10000 |
| Oslo | 22 | 666759 | 3 | 10000 |
| Hedmark | 7 | 196190 | 4 | 5000 |
| Oppland | 5 | 189479 | 3 | 5000 |
| Buskerud | 14 | 279714 | 5 | 10000 |
| **Vestfold** | 18 | 247048 | 7 | 20000 |
| **Telemark** | 24 | 173307 | 14 | 25000 |
| **East Agder** | 30 | 116673 | 26 | 25000 |
| **West Agder** | 38 | 155490 | 24 | 25000 |
| **Rogaland** | 46 | 472024 | 10 | 25000 |
| **Hordaland** | 61 | 519963 | 11 | 25000 |
| **Sogn og Fjordane** | 17 | 110266 | 15 | 20000 |
| **Møre og Romsdal** | 51 | 266274 | 19 | 25069 |
| Sør-Trøndelag | 26 | 317363 | 8 | 10054 |
| Nord-Trøndelag | 4 | 137233 | 3 | 4877 |
| Nordland | 2 | 242866 | 1 | 5000 |
| Troms | 1 | 165632 | 1 | 5000 |
| Finnmark | 0 | 76149 | 0 | 5000 |

* High-endemic counties are shown in bold. The table shows incident LB cases in 2017, the year the sample was planned. The randomly selected sample of adults (aged ≥18 years) was drawn from the National Population Register (NPR) in October 2019, based on incident LB cases over time from MSIS and the population per county. Due to practical and technical aspects of the extraction, actual sample sizes include all available personss aged ≥18 in each county, slightly deviating from planned rounded numbers (e.g., 25,069 vs. 25,000). These minor differences are unlikely to affect the analyses.

| **Table S2**  **Comorbid diseases and concomitant medication** for Norwegians reporting persistent health complaints attributed to tick bites or tick-borne diseases | | | | |
| --- | --- | --- | --- | --- |
|  | The SMS-cohort | High endemic regions | Low endemic regions | *p*-value |
| Neurological disease | 42 (12.9) | 33 (12.2) | 9 (16.4) | 0.404 |
| Rheumatologic disease | 77 (23.7) | 65 (24.1) | 12 (21.8) | 0.720 |
| Endocrinological disease | 33 (10.2) | 26 (9.6) | 7 (12.7) | 0.488 |
| Psychiatric disease | 40 (12.3) | 31 (11.5) | 9 (16.4) | 0.315 |
| Cardiovascular disease | 24 (7.4) | 17 (6.3) | 7 (12.7) | 0.151 |
| ME/CFS | 47 (14.2) | 40 (14.7) | 7 (12.3) | 0.641 |
| COPD / asthma | 26 (7.9) | 23 (8.4) | 3 (5.4) | 0.591 |
| Cancer | 18 (5.5) | 14 (5.1) | 4 (7.3) | 0.516 |
| Dermatological disease | 34 (10.2) | 28 (10.1) | 6 (10.5) | 0.931 |
| Ophthalmological disease | 21 (6.4) | 18 (6.6) | 3 (5.5) | 1.000 |
| Allergies | 58 (17.3) | 47 (16.8) | 11 (19.6) | 0.614 |
| Other diseases | 78 (23.6) | 60 (21.9) | 18 (32.1) | 0.100 |
| One or more concomitant medications | 273 (84.3) | 229 (85.1) | 44 (80.0) | 0.341 |
| More than one comorbid disease | 205 (63.1) | 168 (62.2) | 37 (67.3) | 0.479 |

Continuous variables are presented as mean with 95% confidence intervals and group variables as numbers and percent (%). Statistical analyses with chi-square, Student's t test and Fisher’s exact test and its *p*-values.

| **Table S3** – The estimated weighted prevalences (per 100.000) and weighted seroprevalences of Bb-IgG (%) with estimated 95% CI by the different counties. Adjusted for age and sex. Comparison with the national prevalence. | | | | |
| --- | --- | --- | --- | --- |
|  | Prevalence | *p*-value* | Seroprevalence | *p*-value** |
| \| Norway \| \| --- \| | 122 (109-136) | NA | 36.8 (31.5-42.0) | NA |
| Østfold | 147 (87-208) | 0.115 | 49.1 (26.0-72.1) | 0.543 |
| Akershus | 159 (115-203) | **0.004** | 31.1 (15.6-46.6) | 0.231 |
| Oslo | 104 (69-139) | 0.919 | 48.5 (33.0-64.1) | 0.275 |
| Hedmark | 78 (24-131) | 0.367 | 44.0 (16.3-71.7) | 0.809 |
| Oppland | 58 (11-106) | 0.140 | 64.7 (37.4-91.9) | 0.115 |
| Buskerud | 69 (27-112) | 0.161 | 19.1 (0.9-37.2) | 0.062 |
| ***Vestfold*** | 191 (116-266) | **0.004** | 44.2 (19.4-69.1) | 0.756 |
| ***Telemark*** | 177 (90-263) | **0.041** | 47.1 (18.1-76.1) | 0.645 |
| ***East-Agder*** | 179 (73-286) | 0.084 | 61.8 (26.5-97.0) | 0.274 |
| ***West-Agder*** | 189 (94-285) | **0.025** | 44.0 (13.6-74.3) | 0.809 |
| ***Rogaland*** | 183 (129-237) | **<0.001** | 33.0 (15.4-50.6) | 0.405 |
| ***Hordaland*** | 133 (89-177) | 0.162 | 39.0 (22.2-55.7) | 0.854 |
| ***Sogn og Fjordane*** | 138 (42-233) | 0.459 | 39.7 (3.3-76.1) | 0.960 |
| ***Møre og Romsdal*** | 161 (95-228) | **0.046** | 47.9 (24.2-71.5) | 0.518 |
| Sør-Trøndelag | 81 (37-125) | 0.313 | 34.8 (15.0-54.6) | 0.596 |
| Nord-Trøndelag | 20 (0-53) | **0.04** | 0 (0-97.5) | 0.489 |
| Nordland | 20 (0-44) | **0.005** | 0 (0-97.5) | 0.489 |
| Troms | 60 (8-112) | 0.186 | 0 (0-70.8) | 0.231 |
| Finnmark | 20 (0-64) | 0.135 | 0 (0-97.5) | 0.489 |

* *p*-value for the prevalence. ** *p*-value for the seroprevalence. All comparisons to Norway.

| **Table S4** – Total study population: Linear regression categorized on medical records submitted or not*. Unadjusted and adjusted by age ** and sex | | | | |
| --- | --- | --- | --- | --- |
|  | PHQ-15 | | FSS | |
| Parameters | Unadjusted | Adjusted | Unadjusted | Adjusted |
| constant | 11.9 | 11.7 | 5.2 | 5.2 |
| *b* 95% CI | -1.9 (-3.1, -0.6) | -1.2 (-2.4, 0.04) | -0.37 (-0.7, -0.02) | -0.3 (-0.7, 0.06) |
| Standardized *β* | -0.151 | -0.1 | -0.10 | -0.08 |
| *p*-value | **0.004** | 0.058 | **0.038** | 0.105 |
|  | HAD depression | | HAD anxiety | |
| Parameters | Unadjusted | Adjusted | Unadjusted | Adjusted |
| constant | 5.3 | 4.6 | 5.9 | 5.1 |
| *b* 95% CI | -0.79 (-1.5, -0.03) | -0.73 (-1.5, 0.1) | -0.92 (-1.7, -0.12) | -0.94 (-1.8, -0.1) |
| Standardized *β* | -0.1 | -0.1 | -0.11 | -0.11 |
| *p*-value | **0.043** | 0.075 | **0.024** | **0.027** |
|  | PCS | | MCS | |
| Parameters | Unadjusted | Adjusted | Unadjusted | Adjusted |
| constant | 38.2 | 36.8 | 44.8 | 46.2 |
| *b* 95% CI | 1.9 (0.23, 3.6) | 1.8 (0.01, 3.6) | 1.1 (-0.53, 2.8) | 1.2 (-0.5, 3.0) |
| Standardized *β* | 0.11 | 0.10 | 0.07 | 0.07 |
| *p*-value | **0.026** | **0.049** | 0.181 | 0.170 |

Reference group (*) includes participants who did not submit medical records. The unstandardized coefficient (*b*) represents the difference for those who submitted medical records compared to those who did not (reference group), based on age- and sex-adjusted linear regression analyses. Age (**) is categorized as 18–49 and 50+.

| **Table S5** – The SMS-cohort: Linear regression categorized on medical records submitted or not*. Unadjusted and adjusted by age ** and sex | | | | |
| --- | --- | --- | --- | --- |
|  | PHQ-15 | | FSS | |
| Parameters | Unadjusted | Adjusted | Unadjusted | Adjusted |
| constant | 12.2 | 11.9 | 5.1 | 5.2 |
| *b* 95% CI | -2.2 (-3.6, -0.8) | -1.4 (-2.8, -0.05) | -0.31 (-0.7, 0.1) | -0.25 (-0.6, 0.15) |
| Standardized *β* | -0.19 | -0.124 | -0.09 | -0.07 |
| *p*-value | **0.002** | **0.042** | 0.108 | 0.221 |
|  | HAD depression | | HAD anxiety | |
| Parameters | Unadjusted | Adjusted | Unadjusted | Adjusted |
| constant | 5.3 | 4.5 | 6.1 | 5.5 |
| *b* 95% CI | -0.7 (-1.6, 0.12) | -0.65 (-1.6, 0.25) | -1.2 (-2.1, -0.3) | -1.2 (-2.1, -0.32) |
| Standardized *β* | -0.1 | -0.09 | -0.15 | -0.16 |
| *p*-value | 0.092 | 0.155 | **0.009** | **0.009** |
|  | PCS | | MCS | |
| Parameters | Unadjusted | Adjusted | Unadjusted | Adjusted |
| constant | 39.9 | 38.3 | 44.7 | 45.7 |
| *b* 95% CI | 0.24 (-1.3, 1.8) | 0.16 (-1.5, 1.8) | 1.3 (-0.3, 2.9) | 1.5 (-0.26, 3.2) |
| Standardized *β* | 0.02 | 0.01 | 0.09 | 0.10 |
| *p*-value | 0.757 | 0.850 | 0.117 | 0.096 |

Reference group (*) includes participants who did not submit medical records. The unstandardized coefficient (*b*) represents the difference for those who submitted medical records compared to those who did not (reference group), based on age- and sex-adjusted linear regression analyses. Age (**) is categorized as 18–49 and 50+.

| **Table S6** - Linear regression categorized on high- and low* endemic regions. Unadjusted and adjusted by age ** and sex | | | | |
| --- | --- | --- | --- | --- |
|  | PHQ-15 | | FSS | |
| Parameters | Unadjusted | Adjusted | Unadjusted | Adjusted |
| constant | 13.0 | 12.2 | 5.1 | 5.1 |
| *b* 95% CI | -2.2 (-4.1, -0.2) | -1.3 (-3.2, 0.6) | -0.16 (-0.7, 0.4) | 0.04 (-0.6, 0.5) |
| Standardized *β* | -0.131 | -0.08 | -0.03 | -0.01 |
| *p*-value | **0.029** | 0.172 | 0.551 | 0.896 |
|  | HAD depression | | HAD anxiety | |
| Parameters | Unadjusted | Adjusted | Unadjusted | Adjusted |
| constant | 4.9 | 4.1 | 6.6 | 5.9 |
| *b* 95% CI | 0.0 (-1.2, 1.2) | -0.0 (-1.2, 1.2) | -1.3 (-2.5, -0.05) | -1.3 (-2.6, -0.08) |
| Standardized *β* | 0.00 | 0.00 | -0.115 | -0.122 |
| *p*-value | 0.999 | 0.996 | **0.041** | **0.037** |
|  | PCS | | MCS | |
| Parameters | Unadjusted | Adjusted | Unadjusted | Adjusted |
| constant | 38.6 | 37.1 | 45.3 | 46.7 |
| *b* 95% CI | 1.7 (-0.5, 3.8) | 1.6 (-0.7, 3.8) | 0.02 (-2.2,2.2) | -0.11 (-2.4, 2.2) |
| Standardized *β* | 0.09 | 0.08 | 0.001 | -0.05 |
| *p*-value | 0.126 | 0.166 | 0.988 | 0.928 |

Reference group (*) includes participants from low endemic regions. The unstandardized coefficient (*b*) represents the difference for those from high endemic regions compared to those from low endemic regions (reference group), based on age- and sex-adjusted linear regression analyses. Age (**) is categorized as 18–49 and 50+.

| **Table S7 -** PROMs and normative data * | | | | | | |
| --- | --- | --- | --- | --- | --- | --- |
|  | PHQ-15 | | PCS | | FSS | |
|  | Cases | Normal | Cases | Normal | Cases | Normal |
| Number (n) | 339 | 3406 | 336 | 4861 | 339 | 1859 |
| Mean | 11.1 | 6.3 | 38.7 | 42.3 | 5.0 | 4.0 |
| Standard deviation | 5.9 | 4.6 | 8.9 | 9.3 | 1.76 | 1.31 |
|  | HAD-D | | HAD-A | |  |  |
|  | Cases | Normal | Cases | Normal |  |  |
| Number (n) | 384 | 39573 | 384 | 39277 |  |  |
| Mean | 5.02 | 3.33 | 5.37 | 4.02 |  |  |
| Standard deviation | 3.84 | 2.89 | 3.98 | 3.29 |  |  |

* The same age groups were used for comparisons between cases and normative data,

but different groups were used for each outcome variable due to varying age groups in

the normative data: PHQ-15 (age 18-79 years), PCS (age 20-79 years),

FSS (age 18-80 years). The age range for the HAD score is not defined in the normative data. All differences were statistically significant (*p*<0.001).

| **Table S8** – Clinical data from medical records categorized by endemic regions. | | | | |
| --- | --- | --- | --- | --- |
|  | Proportions (%) | High-endemic  (N, %) | Low-endemic  (N, %) | *p*-value |
| More than 12 months since antibiotic treatment against LD | 16/83 (19.3) | 16 (21.1) | 0 (0) | 0.336 |
| Latency for treatment >= 6 weeks | 12/53 (22.6) | 11 (25.0) | 1 (11.1) | 0.665 |
| Verified LB treated* | 102/169 (60.4) | 91 (61.1) | 11 (55.0) | 0.602 |
| Two or more treatments for LB | 38/144 (26.4) | 33 (22.1) | 5 (25.0) | 0.778 |
| Treatment four weeks or more (LB) | 28/169 (16.6) | 23 (15.4) | 5 (25.0) | 0.334 |
| Verified tick-borne disease prior to persistent symptoms | 14/169 (8.3) | 11 (7.4) | 3 (15.0) | 0.219 |
| Verified tick-bite GP | 59/169 (34.9) | 51 (34.2) | 8 (40.0) | 0.611 |
| EM or Lymphocytoma | 47/169 (27.8) | 44 (37.6) | 3 (21.4) | 0.233 |
| Disseminated borreliosis** | 38/169 (22.5) | 32 (21.5) | 6 (30.0) | 0.399 |

*Even if LB not verified, they can still have received antibiotic treatment against unverified LB. ** Stadium 2 and/or 3.

| **Table S9** – Ongoing symptoms and cognitive difficulties reported in medical journals possibly attributed to Lyme disease (N=169, given %) | |
| --- | --- |
| ***Symptoms attributed to Lyme disease*** |  |
| Fatigue | 33 (19.5) |
| Musculoskeletal issues | 26 (15.4) |
| Pain (anatomic locations below) | 18 (10.7) |
| *Leg* | 10 (5.9) |
| *Arm, shoulder or back* | 8 (4.7) |
| *Stomach* | 2 (1.2) |
| *Chest* | 1 (0.6) |
| *Pelvis* | 1 (0.6) |
| Headache | 17 (10.1) |
| Ataxia | 5 (3.0) |
| Vertigo | 4 (2.4) |
|  |  |
| ***Neurocognitive difficulties attributed to Lyme disease*** |  |
| Consentration | 10 (5.9) |
| Memory | 4 (2.4) |

| **Table S10** – Medical records – The type of antibiotic treatment on LB. Proportions (%). | | | | | | |
| --- | --- | --- | --- | --- | --- | --- |
|  | Doxycycline (p.o.) | Penicilline (p.o.) | Penicilline (i.v.) | Ceftriaxone (i.v.) | Antibiotic treatment not specified | Antibiotics in combination |
| Verified and treated LB | 74/102 (72.5) | 29/102  (28.4) | 8/102  (7.8) | 7/102  (6.9) | 4/102  (3.9) | 4/102  (3.9) |
| Verified tick-borne disease prior to onset of persistent symptoms | 5/14  (35.7) | 6/14  (42.9) | 0 | 1/14  (7.1) | 1/14  (7.1) | 1/14  (7.1) |
| EM or Lymphocytoma | 17/47  (36.2) | 12/47  (25.5) | 2/47  (4.3) | 1/47  (2.1) | 2/47  (4.3) | 2/47  (4.3) |
| Disseminated borreliosis* | 24/38  (63.2) | 8/38  (21.1) | 5/38  (13.2) | 3/38  (7.9) | 3/38  (7.9) | 2/38  (5.3) |
| Neuroborreliosis verified by lumbar puncture | 12/17  (70.6) | 3/17  (17.6) | 0 | 2/17  (11.8) | 2/17  (11.8) | 1/17  (5.9) |

*Stadium 2 and 3. The denominator is the first column listing the different manifestations of tick-borne diseases, while the numerator is the different antibiotics mentioned in the first row.

| **Table S11** – Medical records: Proportions with improvement after antibiotic treatment on LB | | | | | | |
| --- | --- | --- | --- | --- | --- | --- |
|  | Verified and treated LB  (*p*-value) | Not verified LB, but treated ex juvantibus  (*p*-value) | Verified tick-borne disease prior to persistent symptoms  (*p*-value) | EM or Lymphocytoma*  (*p*-value) | Disseminated borreliosis*  (*p*-value) | Neuroborreliosis verified by lumbar puncture*  (*p*-value) |
| Doxycycline (p.o.) | 36/45 (80%)  0.031 | 2/6 (33.3%)  0.031 | 5  (100%)  NA | 9/11  (81.8%)  NA | 10/13  (76.9%)  NA | 6/7  (85.7%)  NA |
| Penicillin (p.o.) | 19/20 (95%)  NA | NA | 4  (100%)  NA | 8  (100%)  NA | 4  (100%)  NA | 1  (100%)  NA |
| Ceftriaxone (i.v.) | 5/6  (83.3%)  NA | NA | 1  (100%)  NA | 1  (100%)  NA | 2  (100%)  NA | 1  (100%)  NA |
| Antibiotic treatment not specified | 2/4  (50%)  0.467 | 2  (100%)  0.467 | 1  (100%)  NA | 2  (100%)  NA | 2  (100%)  NA | 2  (100%)  NA |
| Antibiotics in combination | 3  (100%)  NA | 1  (100%)  NA | 1  (100%)  NA | 2  (100%)  NA | 1  (100%)  NA | 1  (100%)  NA |

*Reference category is borreliosis not proven and no improvement or worsening of symptoms. Given per cent (%). NA means not applicable.

### **Supplementary documentation on serological and molecular diagnostics**

### *Serology*

We received blood samples from 385/470 (81.9%) of the included persons. Serum IgG antibodies against *Bb* sensu lato were measured using the enzyme-linked immunosorbent assay kit Enzygnost® Lyme link VIsE/ IgG (ELISA) (Siemens Healthcare Diagnostics Products GmbH, Erlangen, Germany) (subjects recruited by general practitioners and by invitation). This test was no longer available (discontinued by the manufacturer) when we received sera from the SMS-study; therefore, we switched to the Serion ELISA classic *Bb* IgG (subjects from SMS recruitment). Parallel examination of serum panel between these two kits revealed excellent agreement. The cut off limit for IgG antibodies for *Bb* was set to >5 U/ml. Samples with an equivocal score were classified as negative. IgG antibodies against TBEV, *F. tularensis*, and *C.* *burnetii* phase 2 antigen were analysed with SERION ELISA classic kits (Serion Diagnostics, Institut Virion/ Serion GmbH, Wurzburg, Germany) according to the manufacturer's instructions. The classification of sera as negative, equivocal, and positive was performed according to the kit instructions. Indirect immunofluorescent assay (IFA) tests were used for the detection of serum IgG antibodies against *Anaplasma phagocytophilum* (*Anaplasma phagocytophilum* IFA IgG), *Babesia microti* (*Babesia microti* IFA IgG), *Bartonella henselae* and *quintana* (*Bartonella* IFA IgG) from Focus Diagnostics of Cypress, California, USA, and *Rickettsia helvetica* and *conorii* (*Rickettsia* Screen IFA IgG Antibody Kit) and *Babesia divergens* (*Babesia divergens* IgG IFA Kit) from Fuller Laboratories, Fullerton, California, USA. Due to substantial cross-reactivity for IgG antibodies within *Bartonella henselae/quintana* and within the *Rickettsia helvetica/conorii*, the results are summarised for the two *Bartonella* species and the two *Rickettsia* species. Analyses and interpretation of results were performed according to the manufacturer's instructions. A screening dilution of 1:64 was applied for the evaluation of IFA tests. The IFA slides were evaluated separately by two investigators (only one investigator for the SMS cohort). Positive sera were titrated further to give an end titre. *Bartonella* and *Coxiella* were included due to public awareness that these microbes could be potential tick-borne agents, despite no established link to tick bites. This protocol for serological methods has been published previously [1].

### *Preparation of DNA samples*

All DNA preparation were performed at the Department of Medical Microbiology at Sørlandet Hospital (Kristiansand, Norway). DNA was isolated using the MagNAPure 96 DNA and viral NA small volume kit (Roche, Mannheim, Germany) or by QIAamp DNA Mini Kit (Qiagen,Venlo, The Netherlands) from 200 µl of whole blood and the plasma/buffy coat fractions of patient EDTA blood. Plasma/buffy coat was collected after centrifugation at 1000x g for 12 min and concentrated at 200 µl by centrifugation at 10,000x g for 2 min. DNA from both fractions (n=369) or the whole blood fraction (n=12) were successfully extracted from fresh material, while frozen whole blood was used for the rest (n=5). The DNA of the patients was analysed after storage at -20°C.

### *Real-time PCR*

Real-time PCR protocols (PCR no. 1-11 in **Table S12**) were performed at Department of Medical Microbiology at Sørlandet hospital (Kristiansand, Norway, by HQ). Samples from 101 patients (recruited by invitation or GP) were analysed by PCR no. 1-6 and 8-10 in August 2018. Samples from 285 patients recruited by SMS were analysed by PCR no. 1-5 and 7-11 in May-June 2021. Each protocol used 5 µl of DNA in a 15 µl reaction mixture consisting of 5 mM MgCl_2,_ 0.5 units uracil DNA-glycosylase (Eurogentec S.A. Seraing, Belgium), and LightCycler FastStart DNA master mix (Roche) with primers and probe(s) in the concentrations given in supplementary table. Real-time assays detecting *B. burgdorferi* s.l., *B. miyamotoi*, *Rickettsia* spp*., N. mikurensis* (CNM-II), F. tularensis, Bartonella spp., C. burnetii and the three *Babesia* spp. (triplex PCR) were run on a LightCycler (LC) 480 with the following thermocycling parameters; 2 min at 40°C followed by 10 min at 95°C and 47 cycles of 15 s at 95°C, 30 s at 60°C and 20 s at 72°C. The following PCR program was used for running the protocol detecting *A. phagocytophilum* on a LC 2.0; 2 min at 40°C followed by 10 min at 95°C and 55 cycles of 5 s at 95°C, 10 s at 55°C and 5 s at 72°C. The program for analytical melting was 5 s at 95°C, 2 min at 40°C and an increase to 80°C at a 0.2°C/s ramp rate. The amplification parameters used for the CNM-I assay detecting *N. mikurensis* run on LC 1.0 were as following; 2 min at 40°C followed by 10 min at 95°C and 45 cycles of 15 s at 95°C, 60 s at 54°C. Patient samples (two fractions each patient) were analysed once by each real-time PCR. Positive samples were re-tested in triplicates with single PCR and confirmed positive if at least one of the reactions were positive.

To rule out the presence of nontypical *Babesia* species, the samples from 101 patient recruited by invitation or GP were sent to another laboratory (the Norwegian Veterinary Institute, by ØØ) for additional *Babesia* screening, except for two buffy coat samples (one out of two fractions for two of the patients) which were unavailable due to depletion. The aliquoted samples were further checked using a previously described real-time PCR setup (PCR no. 11). Additionally, a newly designed real-time PCR (PCR no. 12) was employed to the remaining samples (except one plasma fraction that was depleted after first PCR run) for the detection of small *Babesia* spp. parasites that were not detectable by the BdiF/BdiR and BdiT primer/probe set. Assay was designed using an alignment of 18S *Babesia* sequences from GenBank, with the objective of detecting *Bab*. *gibsoni* and *Bab. microti*. PCR was performed in 25 µL reaction mix using 1x qPCR Brilliant III Probe Master Mix (Agilent Technologies, Santa Clara, USA) and run on a BioRad CFX96 real-time PCR instrument (Hercules, USA) using the manufacturer’s two-step FAST PCR protocol, running up to 40 cycles. Positive control DNA from *Bab. microti* and *Bab. gibsoni* yielded signals, but not for *Bab. divergens*, confirming the specificity of the assay (data not presented). Each run included a negative control and a *Bab. microti* positive control. No patient samples tested positive. To further ensure that other species of *Babesia* spp. were not present, all patient samples were subjected to standard PCR (PCR no. 13) These primers have been used in research to identify a variety of *Babesia* spp. in various tick and animal blood samples (Øines, unpublished material). The PCR was carried out under non-stringent conditions (annealing temperature 50°C, up to 37 cycles) to further allow amplification of any parasite DNA, if present. This PCR has the potential to amplify non-specific DNA, alongside *Babesia* targets, so to rule out false positive, gel electrophoresis of the products would enable visual confirmation of the PCR products that could be indicative of *Babesia* DNA. A *Bab. divergens* positive control was included in the run as a visual reference. Inspection of the gel revealed, only one band per sample corresponding to the host DNA. This also confirmed that DNA was present in the samples, but did not confirm any parasite DNA presence. To rule out the small chance that any of these products were of parasite origin, a subset of these PCR products was subjected to Sanger sequencing using the same PCR primers. Sequencing results confirmed the absence of *Babesia* DNA, with all signals indicating DNA from the host genome. The analyses were performed between August and September 2019.

*Validation of assays*

Assays 1–11 and 12–13 were performed at the National Reference Laboratory for Borrelia diagnostics in Norway and at the Norwegian Veterinary Institute, respectively. The two PCR assays for detection of *Borrelia burgdorferi* sensu lato have been in routine diagnostic use since 2008, and their analytical performance has been validated in published studies and through participation in external quality assessment programs [2]. Both assays detect *B. burgdorferi* s.s., *B. afzelii*, *B. garinii*, *B. bavariensis*, *B. bissettii*, *B. valaisiana* and *B. mayonii*. The 16S rRNA assay additionally detects *B. spielmanii* and may cross-react with relapsing fever *Borrelia* species. Neither assay detects *B. japonica* or *B. lusitaniae*.

The two PCR assays for *N. mikurensis* are validated for human diagnostics and in routine use: one at the National Reference Laboratory for *N. mikurensis* at Sahlgrenska University Hospital, Sweden (CNM-I), and the other at the National Reference Laboratory for Borrelia diagnostics, Sørlandet Hospital, Norway (CNM-II). Both assays have shown concordant results [3, 4]. The CNM-II assay has also been tested against *Anaplasma phagocytophilum*, *Midichloria mitochondrii*, *Wolbachia*, *Ehrlichia canis*, *E. chaffeensis* and *E. muris*, with no cross-reactions detected [5].

The *Babesia* assays used in this study have not been formally compared against each other but have been applied in various research projects, including analyses of tick DNA, verification of positive controls, and confirmation of clinical Babesiosis cases from animal tissues and blood samples. These applications have demonstrated the robustness of the assays.

**Table S12 -** Characteristics and sequences of primers and probes used in this study

| PCR  no | Agents  detected  by PCR | Names  Primers and probes | Target  gene | Oligonucleotide sequence (5’-3’) | Concentration of primers/  probes (µM) | Refer-ence |
| --- | --- | --- | --- | --- | --- | --- |
| *1* | *Borrelia*  *burgdorferi* | *-* | *ospA* | *F* ATATTTATTGGGAATAGGTCTAATAT  *R* CTTTGTCTTTTTCTTTRCTTACAAG  *P*  AAGCAAAATGTTAGCAGCCTTGA | 0.5/0.4 | [6] |
| *2* | *Borrelia*  *burgdorferi* | - | 16S rRNA | *F* GCTGTAAACGATGCACACTTGGT  *R* GGCGGCACACTTAACACGTTAG  *P* TTCGGTACTAACTTTTAGTTAA-MGB | 0.5/0.2 | [7] |
| *3* | *Borrelia miyamotoi* | - | 16S rRNA | *F* GCTGTAAACGATGCACACTTGGT  *R*  GGCGGCACACTTAACACGTTAG  *P* CGGTACTAACCTTTCGATTA-MGB | 0.5/0.2 | [7] |
| *4* | *Anaplasma*  *phagocytophilum* | - | groESL | *F* AAGACGAAATTGCACAAGT  *R* AGCCTTTGCTTTCTTCAAC  *FL* CTTTAACACACTGTGCAATCTTACTTCC  *LC* TGTTCTTGTCTCCATTCGCAG | 0.5 (F)/  0.2 (FL/LC) | [8] |
| *5* | *Rickettsia*  *SFG* and *TG* | - | gltA | *F* TCGCAAATGTTCACGGTACTTT  *R* TCGTGCATTTCTTTCCATTGTG  *P* TGCAATAGCAAGAACCGTAGGCTGGATG | 0.2/0.2 | [9] |
| *6* | Neoehrlichia mikurensis (CNM-I) | - | groEL | *F* CGGAAATAACAAAAGATGGA  *R* ACCTCCTCGATTACTTTAG  *P* TTGGTGATGGAACTACA-MGB | 1.0/0.2 | [10] |
| *7* | *Neoehrlichia mikurensis*  (CNM-II) | - | groEL | *F* GCAAATGGAGATAAAAACATAGGTAGTAAA  *R* CATACCGTCAGTTTTTTCAACTTCTAA  *P* TTACAGTTGAGGAAAGTAAGGGA-MGB | 0.5/0.2 | [5] |
| *8* | *Francisella*  *tularensis* | - | fopA | *F* GGCAAATCTAGCAGGTCA  *R* GCTGTAGTCGCACCATTATC  *FL* ATGGCAGAGCGGGTACTAACATGATTG  *LC* TGCTGGTTTAACATGGTTCTTTGGTGG | 0.5/  0.2 (Fl)  0.4 (LC) | [11] |
| *9* | *Bartonella* | - | CS | *F* GGGGACCAGCTCATGGTGG  *R* CGTGGATCATAATTTTTATA  *P* CCAAAACCCATAAGGCGGAAAGGATCATTT | 0.2 (F)  0.6 (R)/  0.1 | [12] |
| *10* | *Coxiella*  *burnetii* | - | icd | *F* CGTTATTTTACGGGTGTGCCA  *R*  CAGAATTTTCGCGGAAAATCA  *P* CATATTCACCTTTTCAGGCGTTTTGACCGT-TAMRA-T | 0.3/0.1 | [13] |
| *11* | *Babesia microti* | - | CCTeta | *F* ACAATGGATTTTCCCCAGCAAAA  *R* GCGACATTTCGGCAACTTATATA  *P* TACTCTGGTGCAATGAGCGTATGGGTA | 0.5/0.2 | [14] |
|  | *Babesia divergens* |  | hsp70 | *F* CTCATTGGTGACGCCGCTA  *R* CTCCTCCCGATAAGCCTCTT  *P* AGAACCAGGAGGCCCGTAACCCAGA | 0.5/0.2 |  |
|  | *Babesia venatorum* |  | 18S rRNA | F GCGCGCTACACTGATGCATT  R CAAAAATCAATCCCCGTCACG  P CATCGAGTTTAATCCTGTCCCGAAAGG | 0.5/0.2 |  |
| *12* | *Babesia divergens*  *Babesia venatorum*  *Babesia capreoli Babesia canis* | BdiF  BdiR  BiT | 18S rRNA | F CAGCTTGACGGTAGGGTATTGG  R TCGAACCCTAATTCCCCGTTA  P FAM-CGAGGCAGCAACGG-MGB | 0.24/0.04 | [15, 16] |
| *13* | *Babesia microti*  *(Babesia gibsoni)* | smallBrtF  smallBrtR  Bamicprobe | 18S rRNA | F GACCTATCAGCTTTGGACGG  R AACCCCAATTCCCCGTGACC  P FAM-TAGGTATTGGCCTACCG-TAMRA | 0.8/0.4 | See *real time PCR* above |
|  |  |  |  |  |  |  |
| *14* | *Babesia* spp. | BTH-1F Babrev | 18S rRNA  PCR/  seq | F CCTGAGAACGGCTACCACATCT  R GAATAATTCACCGGATCACTC | 1 | [17] |
|  |  |  |  |  |  |  |
|  |  |  |  |  |  |  |

Abbreviations: *osp*, outer surface protein; *groESL*, heat shock protein; *glt*, citrate synthase; groEL, heat shock protein; *fop*: *Francisella tularensis* outer membrane protein; CS: citrate synthase; *icd*: isocitrate dehydrogenase; CCTeta, T-complex protein 1 subunit eta; hsp, heat shock protein; MGB, minor groove binder; *F*, forward primer; *R*, reverse primer; *P*, probe; *FL*, fluorescein; *LC*, LightCycler Red640

## **References**

1. Dahlberg, A.O., et al., *Persistent self-reported health complaints in Norwegians who attribute their symptoms to tick bites or tick-borne disease- a cross-sectional controlled study.* BMC Infect Dis, 2025. **25**(1): p. 711.

2. Lager, M., et al., *Molecular detection of Borrelia burgdorferi sensu lato - An analytical comparison of real-time PCR protocols from five different Scandinavian laboratories.* PLoS One, 2017. **12**(9): p. e0185434.

3. Quarsten, H., et al., *Tick-borne Pathogens Detected in the Blood of Immunosuppressed Norwegian Patients Living in a Tick-endemic Area.* Clin Infect Dis, 2021. **73**(7): p. e2364-e2371.

4. Quarsten, H, et al., *Tickborne Neoehrlichia mikurensis in the Blood of Blood Donors, Norway, 2023. Unpublished; accepted for publication in Emerging Infectious Diseases, November 2025.* 2025.

5. Jenkins, A., et al., *Detection of Candidatus Neoehrlichia mikurensis in Norway up to the northern limit of Ixodes ricinus distribution using a novel real time PCR test targeting the groEL gene.* BMC Microbiol, 2019. **19**(1): p. 199.

6. Gooskens, J., et al., *Evaluation of an internally controlled real-time PCR targeting the ospA gene for detection of Borrelia burgdorferi sensu lato DNA in cerebrospinal fluid.* Clin Microbiol Infect, 2006. **12**(9): p. 894-900.

7. Tsao, J.I., et al., *An ecological approach to preventing human infection: vaccinating wild mouse reservoirs intervenes in the Lyme disease cycle.* Proc Natl Acad Sci U S A, 2004. **101**(52): p. 18159-64.

8. Haschke-Becher, E., et al., *First detection of the Anaplasma phagocytophilum groEL-A genotype in man.* J Infect, 2010. **60**(4): p. 300-5.

9. Stenos, J., S.R. Graves, and N.B. Unsworth, *A highly sensitive and specific real-time PCR assay for the detection of spotted fever and typhus group Rickettsiae.* Am J Trop Med Hyg, 2005. **73**(6): p. 1083-5.

10. Grankvist, A., et al., *Infections with Candidatus Neoehrlichia mikurensis and Cytokine Responses in 2 Persons Bitten by Ticks, Sweden.* Emerg Infect Dis, 2015. **21**(8): p. 1462-5.

11. Fujita, O., et al., *Development of a real-time PCR assay for detection and quantification of Francisella tularensis.* Jpn J Infect Dis, 2006. **59**(1): p. 46-51.

12. Ehrenborg, C., et al., *High Bartonella spp. seroprevalence in a Swedish homeless population but no evidence of trench fever.* Scand J Infect Dis, 2008. **40**(3): p. 208-15.

13. Klee, S.R., et al., *Highly sensitive real-time PCR for specific detection and quantification of Coxiella burnetii.* BMC Microbiol, 2006. **6**: p. 2.

14. Michelet, L., et al., *High-throughput screening of tick-borne pathogens in Europe.* Front Cell Infect Microbiol, 2014. **4**: p. 103.

15. Radzijevskaja, J., A. Paulauskas, and O. Rosef, *Prevalence of Anaplasma phagocytophilum and Babesia divergens in Ixodes ricinus ticks from Lithuania and Norway.* International Journal of Medical Microbiology, 2008. **298**: p. 218-221.

16. Øines, Ø., et al., *Prevalence and diversity of Babesia spp. in questing Ixodes ricinus ticks from Norway.* Parasit Vectors, 2012. **5**: p. 156.

17. Zintl, A., et al., *Babesias of red deer (Cervus elaphus) in Ireland.* Vet Res, 2011. **42**(1): p. 7.
